# The chloroplast genome sequences of *Ipomoea alba* and *I. obscura* (Convolvulaceae): genome comparison and phylogenetic analysis

**DOI:** 10.1038/s41598-024-64879-8

**Published:** 2024-06-18

**Authors:** Runglawan Sudmoon, Sanit Kaewdaungdee, Hao Xuan Ho, Shiou Yih Lee, Tawatchai Tanee, Arunrat Chaveerach

**Affiliations:** 1 Faculty of Law, Khon Kaen University, Khon Kaen, 40002 Thailand; 2 Department of Biology, Faculty of Science, Khon Kaen University, Khon Kaen, 40002 Thailand; 3 Faculty of Health and Life Sciences, INTI International University, 71800 Nilai, Negeri Sembilan Malaysia; 4 Faculty of Environment and Resource Studies, Mahasarakham University, Maha Sarakham, 44150 Thailand

**Keywords:** Genetic resources, *Ipomoea*, Morning glory, Phylogenomics, Plastid genome, Evolution, Genetics, Plant sciences

## Abstract

*Ipomoea* species have diverse uses as ornamentals, food, and medicine. However, their genomic information is limited; *I. alba* and *I. obscura* were sequenced and assembled. Their chloroplast genomes were 161,353 bp and 159,691 bp, respectively. Both genomes exhibited a quadripartite structure, consisting of a pair of inverted repeat (IR) regions, which are separated by the large single-copy (LSC) and small single-copy (SSC) regions. The overall GC content was 37.5% for both genomes. A total of 104 and 93 simple sequence repeats, 50 large repeats, and 30 and 22 short tandem repeats were identified in the two chloroplast genomes, respectively. G and T were more preferred than C and A at the third base position based on the Parity Rule 2 plot analysis, and the neutrality plot revealed correlation coefficients of 0.126 and 0.105, indicating the influence of natural selection in shaping the codon usage bias in most protein-coding genes (CDS). Genome comparative analyses using 31 selected *Ipomoea* taxa from Thailand showed that their chloroplast genomes are rather conserved, but the presence of expansion or contraction of the IR region was identified in some of these *Ipomoea* taxa. A total of five highly divergent regions were identified, including the CDS genes *acc*D, *ndh*A, and *ndh*F, as well as the intergenic spacer regions *psb*I-*atp*A and *rpl*32-*ccs*A. Phylogenetic analysis based on both the complete chloroplast genome sequence and CDS datasets of 31 *Ipomoea* taxa showed that *I. alba* is resolved as a group member for series (ser.) Quamoclit, which contains seven other taxa, including *I. hederacea*, *I. imperati*, *I. indica*, *I. nil*, *I. purpurea*, *I. quamoclit*, and *I.* × *sloteri*, while *I. obscura* is grouped with *I. tiliifolia,* both of which are under ser. Obscura, and is closely related to *I. biflora* of ser. Pes-tigridis. Divergence time estimation using the complete chloroplast genome sequence dataset indicated that the mean age of the divergence for Ipomoeeae, Argyreiinae, and Astripomoeinae, was approximately 29.99 Mya, 19.81 Mya, and 13.40 Mya, respectively. The node indicating the divergence of *I. alba* from the other members of *Ipomoea* was around 10.06 Mya, and the split between *I. obscura* and *I. tiliifolia* is thought to have happened around 17.13 Mya. The split between the *I. obscura* accessions from Thailand and Taiwan is thought to have taken place around 0.86 Mya.

## Introduction

*Ipomoea* is the largest genus in Convolvulaceae, containing more than 700 species recorded worldwide^1^. The members of *Ipomoea* are recognized as fast-growing plants with long-trailing stems and are highly adapted to degraded lands, such as newly deposited dunes^2^. In Thailand, there are at least 32 recorded species of *Ipomoea*^1^, and they are known by the locals as ornamental plants, food, and raw materials for traditional medicine production^3,4^. Among these species, *I. alba* and *I. obscura* are species that are commonly found in all areas of Thailand. To date, Thai researchers have given much attention to studies on yield, seed storage techniques, and the natural drug discovery of *I. alba*^5–7^, but only on the seed germination of *I. obscura*^8^. Despite being familiar among Thais, these species are understudied at present.

DNA studies in *Ipomoea* are not a new thing, with the sweet potato (*I. batatas*) being in the limelight as it is a common staple food^9^. DNA characterization of *I. alba* was carried out using the chloroplast DNA restriction site method^10^ and genic simple sequence repeat (SSR) markers^11^, while there is a report on the use of the random amplified polymorphism DNA (RAPD) marker with *I. obscura* included^12^. Phylogenetic studies involving *I. alba* and *I. obscura* were restricted to the use of only the nuclear ribosomal internal transcribed spacer (ITS) region or the nuclear gene *waxy* sequence^13–15^. However, the phylogenetic relationship among *Ipomoea* species using these selected nuclear gene sequences was not well resolved. By considering the disadvantage of short gene sequences in phylogenetic tree reconstruction, a nuclear tree using a subset of single-copy genes was generated and provided significant resolution to these closely related species of *Ipomoea*^16^.

In general, the chloroplast genome is structurally conserved but would undergo rearrangements, including deletions and duplications^17^. Through evolution, the gene content would change due to the expansion and contraction of the inverted repeat (IR) regions^18^. The chloroplast gene sequences are believed to be useful in phylogenetic tree reconstructions in most angiosperms^19^. However, the chloroplast-based phylogenetic tree of Convolvulaceae using four genes, including *rbc*L, *atp*B, *psb*E-*psb*J, and *trn*L-*trn*F, did not yield reliable branch support at the species level^20^. As next-generation sequencing has become more common, the first study of the full chloroplast genome of *Ipomoea* published 28 genomes from 25 species^21^. Since then, other studies have been done on other species in the same genus^22–25^. Chloroplast-based phylogenomic analysis has been demonstrated to provide insights into complicated relationships in many angiosperm families^26^. A revised classification of Ipomoeeae was proposed based on the phylogenetic analysis using the complete chloroplast genome sequences, which contain two major clades, Astripomoeinae and Argyreiinae, as well as seven groups, namely Batatas, Cairica, Obscura, Murucoides, Pes-caprae, Pes-tigridis, and Quamoclit^21^. Although the finding seemed to be quite informed based on previous phylogenetic studies, the limited sample size was not able to warrant a proper classification of *Ipomoea* effectively.

In this study, we sequenced and characterised two Thai *Ipomoea* species, *I. alba* and *I. obscura*, to expand the genomic knowledge of these two understudied species. To infer the phylogenetic relationship within these two species, we included 28 other published chloroplast genomes of *Ipomoea* in the reconstruction of the phylogenetic tree. The study attempts to reveal the chloroplast genome pattern and determine the molecular placement of the two selected *Ipomoea* species.

## Materials and methods

### Plant material

The fresh young leaves of *Ipomoea alba* and *I. obscura* were collected from the natural population in Khon Kaen (16° 28′ 26.5ʺ N 102° 49′ 05.0 ʺ E) and Maha Sarakham (16° 08′ 58.2ʺ N 102° 59′ 07.5ʺ E) provinces, respectively, to use for total genomic DNA extraction. All the experiments were performed following relevant guidelines and regulations. There is no need for permission to collect them because they are common species that grow widely in gardens, fields, forests, or in households. The plants were identified by a proficient botanist, Professor Arunrat Chaveerach, Ph.D. Additionally, the specimens were kept at the Department of Biology, Faculty of Science, Khon Kaen University, under the voucher specimen numbers A. Chaveerach 985.1 and A. Chaveerach 989.1, for *I. alba* and *I. obscura*, respectively.

### DNA extraction

Fresh young leaves of the studied two *Ipomoea* were dried in silica gel beads. The dried leaves were employed for total genomic DNA extraction using the DNeasy Plant Mini Kit (QIAGEN, Germany), based on the manufacturer’s protocol. The integrity of total genomic DNA was detected by 1% agarose gel electrophoresis. DNA purity and quantity were estimated using Qubit™ 4 Fluorometer (Thermo Fisher Scientific, USA).

### Next-generation sequencing, chloroplast genome assembly and annotation

Next-generation sequencing was performed on an Illumina NovaSeq X platform (Illumina, USA). A 350-bp paired-end library was prepared using a TruSeq DNA Sample Prep Kit (Illumina, USA) to obtain 150-bp pair-end reads. The NGS QC Toolkit was used for adapter sequence removal^27^. The chloroplast genome was then assembled using NOVOPlasty v.4.3.5^28^ with the *rbc*L gene of *I. batatas* (GenBank accession no.: MW122507) as the seed sequence. The complete chloroplast genomes were completely annotated using GeSeq v2.03^29^, in which the annotations of the CDS, tRNA, and rRNA are based on the BLAT search and Chloë v0.1.0 functions embedded in the program, and then coupled with manual improvement for the boundaries. The circular chloroplast genome map was illustrated using OGDraw v1.3.1^30^. The two chloroplast genome sequences were deposited at the National Center for Biotechnology Information’s (NCBI) GenBank database (Table 1).

**Table 1:** List of annotated genes in the two *Ipomoea* chloroplast genomes.

Category	Group of function	List of genes
Transcription and translation	Large subunit of ribosome	*rpl*2, *rpl*14, *rpl*16^i^, *rpl*20, *rpl*22, *rpl*23, *rpl*32, *rpl*33, *rpl*36
	Small subunit of ribosome	*rps*2, *rps*3, *rps*4, *rps*7(× 2)^i^, *rps*8, *rps*11, *rps*12, *rps*14, *rps*15(× 2), *rps*16^i^, *rps*18, *rps*19
	DNA-dependent RNA polymerase	*rpo*A, *rpo*B, *rpo*C1^i^, *rpo*C2,
	Ribosomal RNAs	*rrn*4.5(× 2), *rrn*5(× 2), *rrn*16(× 2), *rrn*23(× 2)^i^
	Transfer RNAs	*trn*A-UGC (× 2)^i^, *trn*C-GCA, *trn*D-GUC, *trn*E-UUC, *trn*F-GAA, *trn*M-CAU, *trn*G-GCC, *trn*G-UCC^i^, *trn*H-GUG, *trn*I-CAU(× 2), *trn*I-GAU(× 2)^i^, *trn*K-UUU, *trn*L-CAA(× 2), *trn*L- UAA^i^, *trn*L-UAG, *trn*M-CAU(× 2), *trn*N-GUU(× 2), *trn*P-UGG, *trn*Q-UUG, *trn*R-ACG(× 2), *trn*R-UCU, *trn*S-GCU, *trn*S-GGA, *trn*S-UGA, *trn*T-GGU, *trn*T-UGU, *trn*V-GAC(× 2), *trn*V-UAC^i^, *trn*W-CCA, *trn*Y-GUA
Photosynthesis related genes	Photosystem I	*psa*A, *psa*B, *psa*C, *psa*I, *psa*J
	Photosystem II	*psb*A, *psb*B, *psb*C, *psb*D, *psb*E, *psb*F, *psb*H, *psb*I, *psb*J, *psb*K, *psb*L, *psb*M, *psb*T, *psb*Z
	NADH dehydrogenase	*ndh*A(× 2^a^)^i^, *ndh*B(× 2)^i^, *ndh*C, *ndh*D, *ndh*E, *ndh*F, *ndh*G, *ndh*H(× 2^a^), *ndh*I, *ndh*J, *ndh*K
	Cytochrome b6/f complex	*pet*A, *pet*B^i^, *pet*D^i^, *pet*G, *pet*L, *pet*N
	ATP synthase	*atp*A, *atp*B, *atp*E, a*tp*F^i^, *atp*H, *atp*I
	RubisCO	*rbc*L
	Photosystem assembly factors	*paf*I*, paf*II
	Photosystem biogenesis factor	*pbf1*
Biosynthesis	Maturase	*mat*K
	ATP-dependent protease	*clp*P1^ii^
	Envelope membrane protein	*cem*A
	Acetyl-CoA-carboxylase	*acc*D
	Translational initiation factor 1	*inf*A^o^
	C-type cytochrome synthesis	*ccs*A
Unknown	Hypothetical chloroplast reading frames	*ycf*1(× 2), *ycf*2(× 2)

^i^Comes with one intron.

^ii^Comes with two introns.

^a^Unique to *I. alba.*

^o^Unique to *I. obscura*: (× 2) = comes with duplicates.

### Repeat analyses

The FASTA sequence was uploaded onto MISA-web to identify the simple sequence repeats (SSRs)^31^. The minimum number of repeat parameters was determined at 10, 4, 4, 3, 3, and 3 for mononucleotides, dinucleotides, trinucleotides, tetranucleotides, pentanucleotides, and hexanucleotides. Additionally, the large repeats, which include forward, palindromic, reverse, and complement, were identified with REPuter^32^. The minimum repeat size was set at 30 bp and a Hamming distance of 3. The tandem repeat was analysed with tandem repeat finder v.4.09 with the advanced option^33^.

### PR2 and neutrality plot analyses

Both the Parity Rule 2 (PR2) plot analysis and the neutrality plot analysis were carried out using the annotated genome files of the two *Ipomoea* species as the input data, which were fed into the codon analysis function available on the online website Genepioneer (http://112.86.217.82:9919/#/home). The PR2 plot analysis was conducted based on the relative synonymous codon usage (RSCU) values of the CDS genes in the chloroplast genome. The PR2 values of each gene were calculated using the formulae A_3_ is divided by the sum of A_3_ and T_3_, and G_3_ is divided by the sum of G_3_ and C_3_. If the proportion of G and C (or A and T) is similar, mutation pressure fully influences the codon usage bias. No bias between natural selection and mutation pressure is detected when the value is 0.5. If the genes have a value close to 0.5, the codon bias might be influenced by mutational pressure, while natural selection and other variables would be the factors when the base proportions are too far between A/T and G/C. For the neutrality plot analysis, a scatter plot is constructed, in which the y-axis represents the GC content at the third codon position (GC3) and the x-axis represents the average GC content at the first and second positions of the codon (GC_12_). A regression slope of 0 indicates that natural selection is completely shaping the codon bias. On the other hand, a significant correlation with a slope of 1 means that mutational pressure is shaping the codon usage bias. If the codon usage bias is due to mutation pressure, the regression curve coefficient would be close to or equal to 1; a coefficient close to or equal to 0 would indicate that the codon usage bias is a result of natural selection.

### Inverted repeat border region and genome comparison

The boundary and junction of inverted repeat (IR) regions for the chloroplast genomes of 31 *Ipomoea* taxa from Thailand, including *I. alba* and *I. obscura* obtained from this study, were visualized using the CPJSdraw v1.0^34^. Prior to analysis, based on the availability of genome data, the complete genome sequences of these selected *Ipomoea* taxa that were recorded from Thailand were downloaded from the NCBI GenBank. The list of *Ipomoea* taxa used in this analysis is presented in Supplementary Table S1. By selecting the chloroplast genome sequence of *I. batatas* (GenBank accession no.: MW122507) as the reference genome, the comparative analysis of the two *Ipomoea* chloroplast genome sequences was performed using mVISTA^35^ with Shuffle-LAGAN mode.

### Sequence divergence analysis

The chloroplast genome sequences of the 31 selected *Ipomoea* taxa were aligned using MAFFT v7^36^. The nucleotide diversity (Pi) analysis was carried out using DnaSP v5.10.01^37^, in which the window length was set at 1000 bp and 500 bp for step size. The number of polymorphic and parsimony-informative sites was also calculated.

### Intraspecific variation in *I. obscura*

To investigate the intraspecific differences between the *I. obscura* sampled from Taiwan (GenBank accession no.: LC729554) and the sample used in this study, the complete chloroplast genome sequence of the *I. obscura* from Taiwan was reannotated using GeSeq v2.03 to compare the difference in gene content and structure between the two accessions. Pairwise distance was conducted using MEGA-X^38^ based on the Kimura two-parameter (K2P) nucleotide substitution model using the bootstrap method. A pairwise deletion approach was used for the treatment of missing data or gaps. By using DnaSP v5.10.01, the number of variable sites between the two complete chloroplast genome sequences and their positions in the genome were identified.

### Phylogenetic tree construction

Phylogenetic analysis was conducted using both the complete chloroplast genome sequences and the concatenated dataset of the 76 shared unique CDS of 31 *Ipomoea* taxa recorded in Thailand. Based on the previous finding^39^, three closely related taxa of *Ipomoea* are included as outgroups, including *Hewittia malabarica* (GenBank accession no.: LC729546), *Merremia hederacea* (GenBank accession no.: MZ240749), and *Operculina turpethum* (GenBank accession no.: LC729560). For the complete chloroplast genome sequence dataset, the sequences in the IRa region were excluded from the analysis. Genome sequence alignment was carried out using MAFFT v.7^36^ prior to phylogenetic reconstruction. For the CDS dataset, all the chloroplast genome sequences were reannotated using GeSeq v2.03 to ensure uniformity in gene content. The CDS of each taxon was extracted and aligned using PhyloSuite v.1.2.3^40^ prior to concatenation. The phylogenetic tree of both datasets was reconstructed using two methods: maximum likelihood (ML) and approximate Bayesian inference (aBI) via IQ-tree v1.6.12^41^. The suitable nucleotide substitution model for the dataset was calculated using ModelFinder^42^. Based on the Bayesian inference criterion, the most optimal substitution model for the genome dataset was the transversion model (TVM) with invariant sites (+ I), the default 4 rate categories of the discrete Gamma model (+ G4), and empirical base frequencies (+ F) (= TVM + I + G4 + F). An edge-unlinked partition mode was selected for the concatenated CDS dataset. For the ML tree, a total of 1,000 bootstrap replicates were conducted for both the Shimodaira-Hasegawa approximate likelihood ratio test (SH-aLRT) and ultrafast bootstrap (UFboot) branch supports; aBI was conducted based on the default parameters of IQ-tree. The resulting trees were visualised using FigTree v.1.4.4^43^.

### Divergence time estimation

At present, no reliable fossil records are available for *Ipomoea*. The identity of the single fossil record, *I. menghalayensis*, was seemingly doubtful^44,45^; thus, it was not considered in this analysis. While there is limited information on the estimated divergence time within Convolvulaceae, the nearest identified calibration point to *Ipomoea* would be the root of the most recent common ancestor of *Merremia* and *Operculina*. The age of the branch node was based on the fossil-derived timescale calibrations from TimeTree^46^, which is estimated to be 64 million years ago (Mya). To assign other internal calibrations for the time tree, the complete chloroplast genome sequences of three reference species of Solanaceae were included, i.e. *Nicotiana glauca* (GenBank accession no.: MT985321), *Physalis minima* (GenBank accession no.: PP471919), and *Solanum lasiocarpum* (GenBank accession no.: PP234975). An internal calibration was included for Solanaceae, which was the split between the Nicotianoideae and Solanoideae clades that is estimated to be in the range of confidence interval (CI) = 22.9–44.4 Mya; while the divergence between Convolvulaceae and Solanaceae was estimated to be in the range of CI 59.1–83.8 Mya. Based on the previous finding^47^, Gentianales diverges before Solanales. Therefore, to root the Solanales clade, the complete chloroplast genome sequence of *Coffea arabica* (GenBank accession no.: EF044213) of Rubiaceae was included as an outgroup species. To estimate the divergence time within *Ipomoea*, the ML tree was reconstructed using IQ-tree based on the most optimal substitution model by ModelFinder under the Akaike information criterion, which was TVM + I + F + G4. RelTime analysis was conducted using MEGA X, in which the divergence time estimates for all branching points were computed based on the general-time-reversible (GTR) + I + F + G4 (= GTR + I + F + G4) model. All gaps and missing data were included in the analysis.

## Results

### Chloroplast genome features

**Figure 1:**
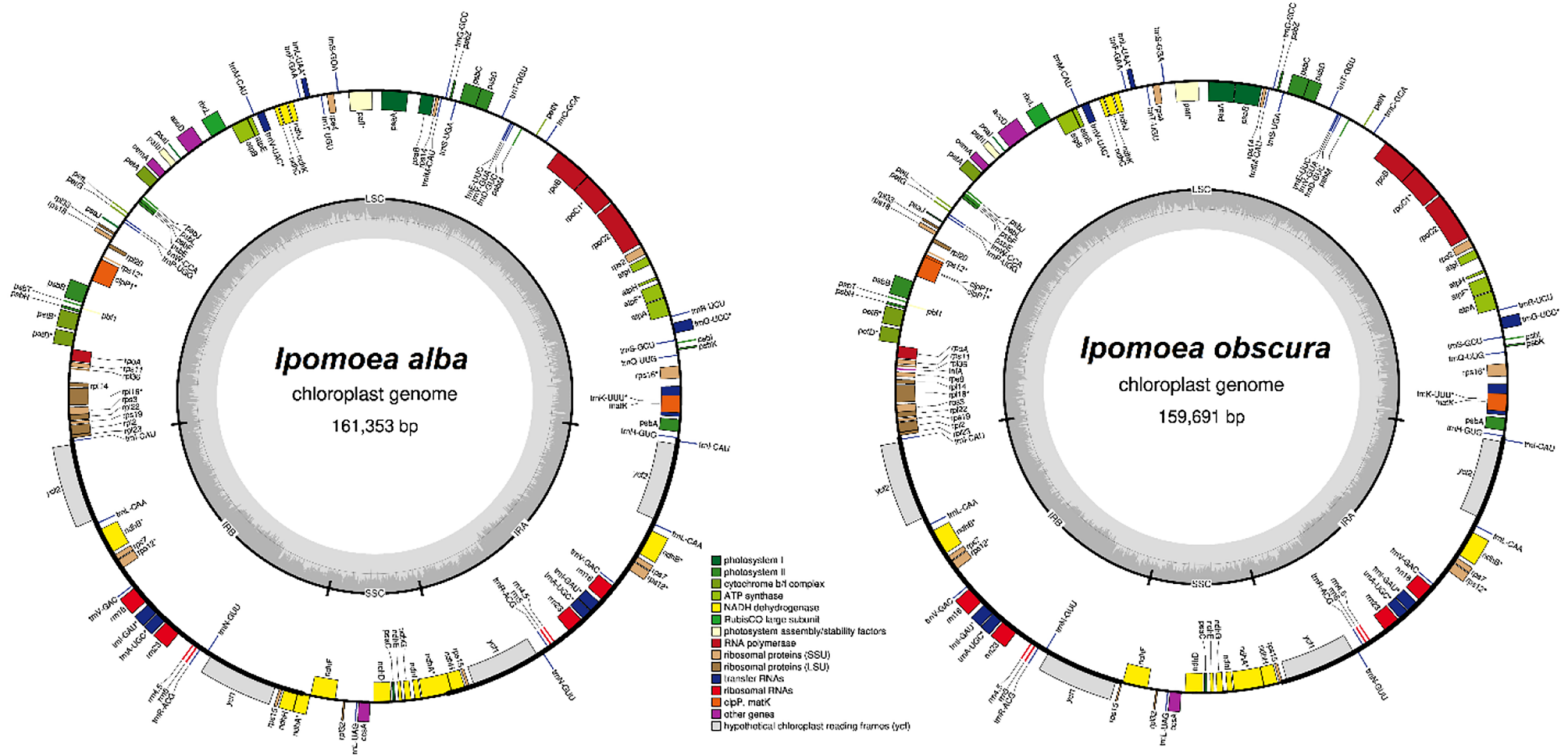
The chloroplast genome map of *Ipomoea alba* (GenBank accession no.: ON209203) and *I. obscura* (GenBank accession no.: OR995405). The genes placed at the outside and inside of the circle are transcribed counterclockwise and clockwise, respectively. Genes are color-coded based on their functional classification. The functional classification of the genes is shown at the centre of the figure.

With a minimum and average sequence depth coverage of 1220 × and 3020 × , as well as 1860 × and 4367 × , for the chloroplast genome assembly of *I. alba* and *I. obscura* (see Supplementary Fig. S1), the chloroplast genome structure of the studied two *Ipomoea* species consisted of a quadripartite structure containing an LSC region, an SSC region, and a pair of IRs (Fig. 1). The genome assembly did not return with any sign of heteroplasmy. The chloroplast genome size of *I. alba* was 161,353 bp, containing 87,848 bp in LSC, 12,075 bp in SSC, and 30,715 bp in IR, while the chloroplast genome size of *I. obscura* was 159,691 bp, containing 88,231 bp in LSC, 13,348 bp in SSC, and 29,056 bp in IR, respectively. The overall GC content was 37.5% in both species, as well as 36.0–36.1%, 32.1–33.2% and 40.7–40.9% for the LSC, SSC, and IR regions of *I. alba* and *I. obscura*, respectively. There were 130 and 129 genes detected in the chloroplast genomes of *I. alba* and *I. obscura*, including 85 and 84 protein-coding (CDS), 37 and 37 tRNA, as well as eight and eight rRNA genes, respectively (Table 1). A total of 16 genes containing introns were identified. The *clp*P1 gene contains two introns, while the other 15 genes, including *atp*F, *ndh*A, *ndh*B, *pet*B, *pet*D, *rpl*16, *rpo*C1, *rps*16, *rrn*23, *trn*A-UGC, *trn*G-UCC, *trn*I-GAU, *trn*K-UUU, *trn*L-UAA, and *trn*V-UAC, contain one intron. The *ndh*A and *ndh*H genes of *I. alba* come in duplicate, but not in *I. obscura*; while the *inf*A gene was only annotated in the chloroplast genome of *I. obscura*, but appears to be unidentified in *I. alba*.

### Sequence repeats

**Figure 2:**
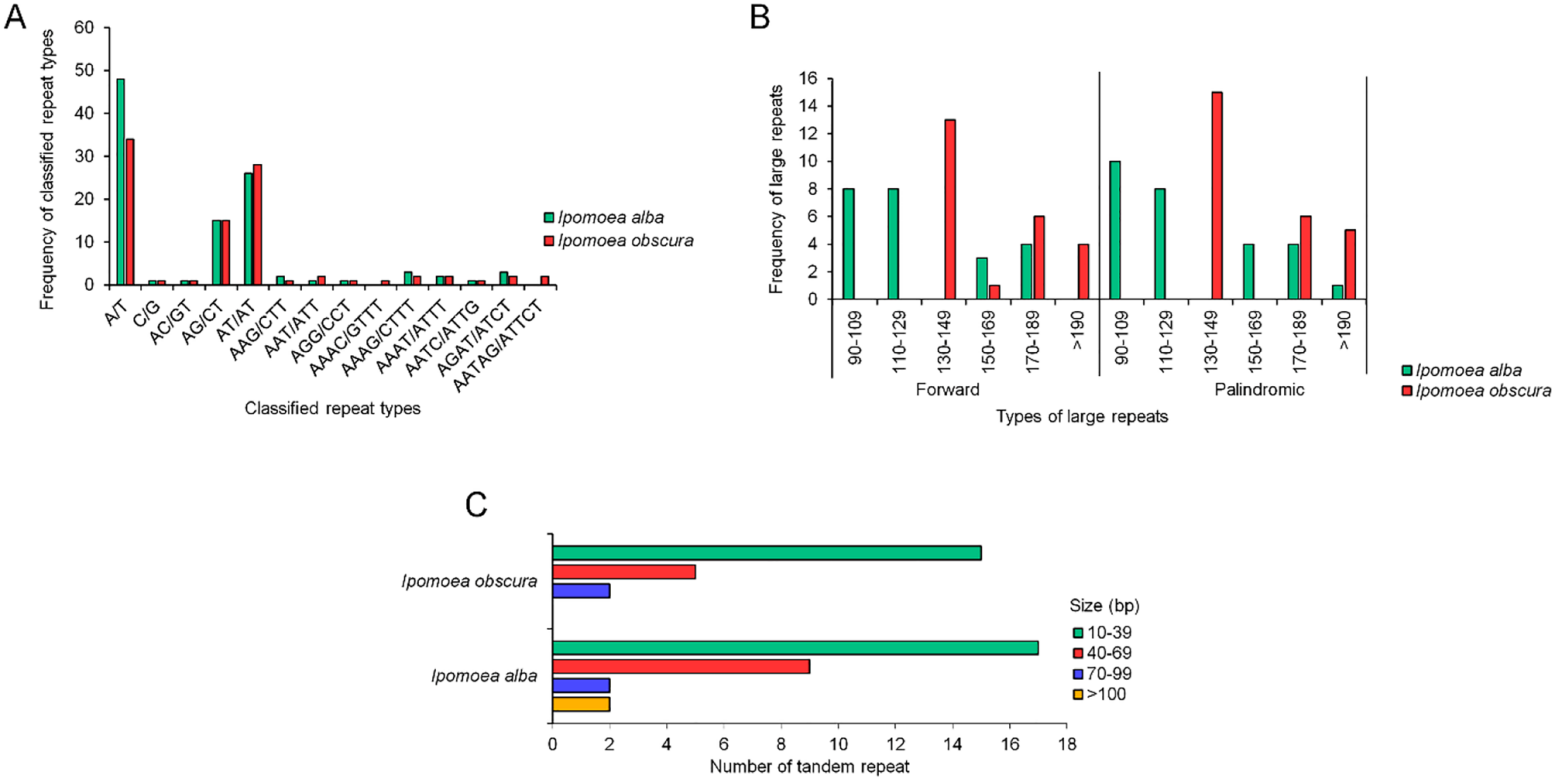
Repeated sequence identification in the two *Ipomoea* chloroplast genomes. (**A**) classified SSR repeat units; (**B**) distribution and frequency of long repeats; (**C**) number of tandem repeats.

A total of 104 and 93 of the SSRs were identified in the *I. alba* and *I. obscura* chloroplast genome sequences, respectively. The most abundant mononucleotide repeat was A/T, detected in 48 and 34 repeats for *I. alba* and *I. obscura*, respectively. For the dinucleotide repeats, the AT/AT type had 26 repeats in *I. alba* and 28 repeats in *I. obscura*. The AG/CT type, on the other hand, had 15 repeats in both species. The trinucleotide repeats as well as the tetranucleotide repeats were detected in both species, except for the AAAC/GTTT repeat type that was not identified in *I. alba*. In addition, the pentanucleotide repeat was only identified in *I. obscura* (Fig. 2A).

A total of 50 large repeats were detected in both species, consisting of only the forward and palindromic repeats (Fig. 2B). The large repeat sizes mostly ranged from 130 to 149 bp. For the tandem repeats, as many as 30 and 22 repeats were identified in *I. alba* and *I. obscura*, respectively. The most repeats were detected within the range of 10 bp to 39 bp for both species (Fig. 2C), while at least two repeats that were more than 100 bp were detected in *I. alba* but not in *I. obscura*.

### Codon usage bias analysis

Based on the RSCU value of the CDS genes of the two *Ipomoea* species (Supplementary Table S2), the PR2 plot for *I. alba* and *I. obscura* showed similar results; the majority values are mainly placed in the region of G3/(G3 + C3) > 0.5 and A3/(A3 + T3) < 0.5 in the analysis (Supplementary Table S3). Overall, the usage frequency of the third base T in the codon is higher than A, and the frequency of G is higher than C. The *rpl* and *rps* genes of both *Ipomoea* species have the tendency to have more As, and in general, photosynthesis-related genes prefer the use of T when compared to self-replication and other genes. Almost all the genes tested showed usage frequencies of G higher than C, except for nine of them, including *atp*I, *cem*A, *ndh*B, *ndh*K, *psb*A, *psb*D, *rpl*14, *rps*3, and *rps*12. Despite the fact that some of the genes had a codon usage bias value close to 0.5, suggesting that mutational pressure could play a role in shaping the bias, many of them were detected to be experiencing natural selection and other variables.

Based on the neutrality plot analysis, the correlation between codon GC12 and GC3 for the chloroplast genomes of the two *Ipomoea* species showed similar trends (Supplementary Fig. S2). The codon GC12 values are recorded between 0.3 and 0.6, while the GC3 values are recorded between 0.1 and 0.4. The regression line slopes for the two *Ipomoea* species ranged from 0.397 to 0.409, with R^2^ = − 0.011–0.016, indicating a negative correlation. As the correlation coefficient, R, would be 0.126 and 0.105 for *I. alba* and *I. obscura*, respectively, this suggests that codon usage bias is a product of natural selection. The GC3 value reflects the percentage of genes affected by mutational pressure, which accounted for approximately 40% to 41% of the total genes, indicating that more than half of the genes are experiencing natural selection in affecting the codon usage bias.

### Comparison of border region and interspecific genome variations

**Figure 3:**
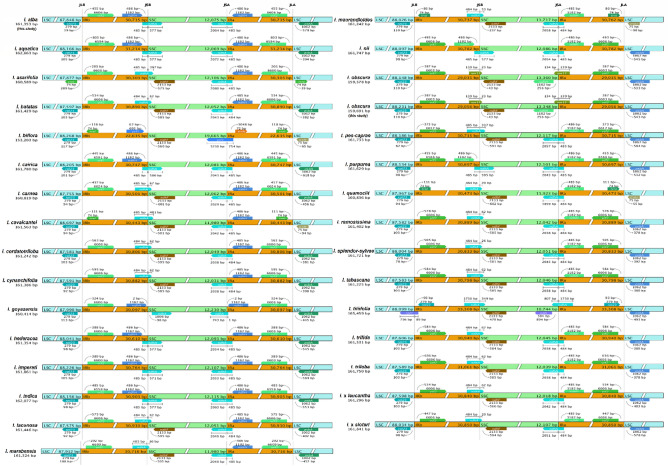
Genes that are located adjacent to the borders of the large single-copy (LSC), small single-copy (SSC), and inverted repeats (IRs) of the chloroplast genomes of 31 *Ipomoea* taxa recorded in Thailand. The boxes below and above the lines indicate the adjacent border gene.

The chloroplast genome sequences of *I. alba* and *I. obscura* revealed different gene content adjacent to the region boundary (Fig. 3). For the junction between LSC and IRb (JLB), both the *rpl*23 and *ycf*2 genes are placed adjacent to the junction in the LSC and IRb regions, respectively, in most taxa, except for *I. biflora*, *I. cavalcantei*, *I. maurandioides*, *I. quamoclit*, which had the *trn*I gene placed in the IRb region. Aside from that, *I. asarifolia* had the *trn*I gene placed in the LSC region, while *I. tiliifolia* had the *rpl*23 gene placed in the IRb region, and the *rpl*2 gene crossed from the LSC into the IRb region. For the junction between SSC and IRb (JSB) and the junction between SSC and IRa (JSA), the *ndh*A genes were detected crossing over from the IR regions into the SSC region in most Ipomoea taxa, except for *I. goyazensis*, where the *ndh*A gene was placed in the SSC region adjacent to JLB but extending into IRa by a 1-bp distance from JSA. Instead of the *ndh*A genes, the *ycf*1 gene was located in the IR regions, with *ycf*1 in the IRa region crossing JSA into the SSC region in *I. biflora*. On the other hand, the two *I. obscura* genomes had the *rps*15 genes crossing from the IR regions into the SSC region. The *ycf*2 and *psb*A genes are respectively located at the IRa and LSC regions, adjacent to the junction between LSC and IRa (JLA) in most *Ipomoea* taxa, while *I. asarifolia* had a *trn*I gene placed in the LSC region instead of the *psb*A gene. However, four *Ipomoea* species, including *I. biflora*, *I. cavalcantei*, *I. maurandioides*, and *I. quamoclit*, revealed a different gene content. The genes adjacent to JLA for these four *Ipomoea* species were the *trn*I and *trn*H genes, which were located in the IRa and LSC regions, respectively.

**Figure 4:**
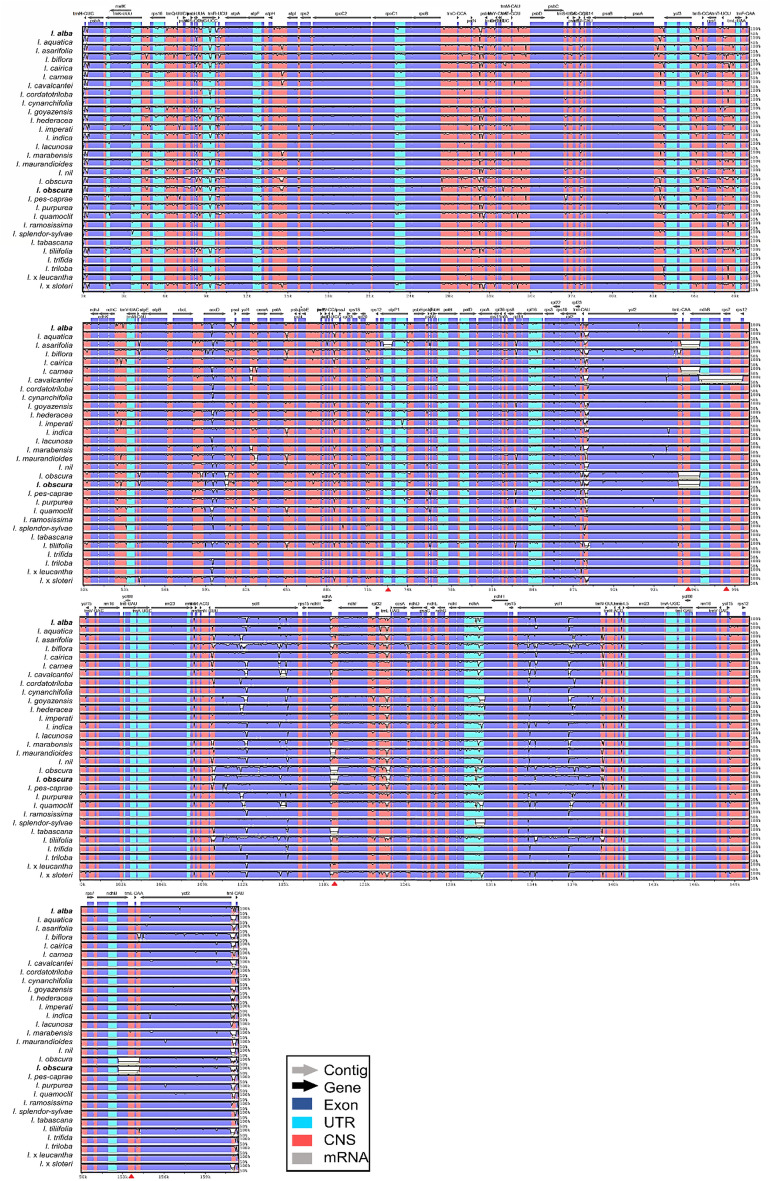
Interspecific chloroplast genome variation of 31 selected *Ipomoea* taxa in Thailand, when compared to *I. batatas* (GenBank accession no.: MW122507) using mVISTA, The purple bars represent exons; the pink bars represent non-coding sequences; the light-blue bars represent tRNA and rRNA regions; the grey arrows above the aligned sequences indicate the genes and their orientations; the x-axis represents the number of bases in the aligned sequences; the y-axis represents the percent identity from 50 to 100%; the dark grey bars indicate variation regions in the genomes; red triangle at the bottom of the x-axis indicates the location of distinct gap in the genome.

Chloroplast genome sequence alignment using Shuffle-Lagan mode revealed similar genome sequence variation among the selected 30 *Ipomoea* taxa when compared to the complete chloroplast genome sequence of *I. batatas* (Fig. 4). At least 12 distinct gaps that represent nucleotide variations are detected in the genome sequence of five *Ipomoea* taxa. In the LSC region, a distinct gap was identified in the *clp*P1 gene of *I. asarifolia*. The following sequence variation was identified at the genes *trn*L-CAA and *ndh*B of the IRb region, as well as their intergenic spacer regions of *I. asarifolia*, *I. carnea*, and in both IR regions for the two *I. obscura* accessions. A large gap was identified stretching from the *ndh*B gene to the *rps*12 gene in the IRb of *I. cavalcantei*. The other four gaps can be found at the SSC region, at the non-coding region next to the 5′ end of the *ndh*F gene of *I. maurandioides*, the two *I. obscura*, and *I. tabascana*.

### Polymorphic sites and nucleotide hotspot recognition

**Figure 5:**
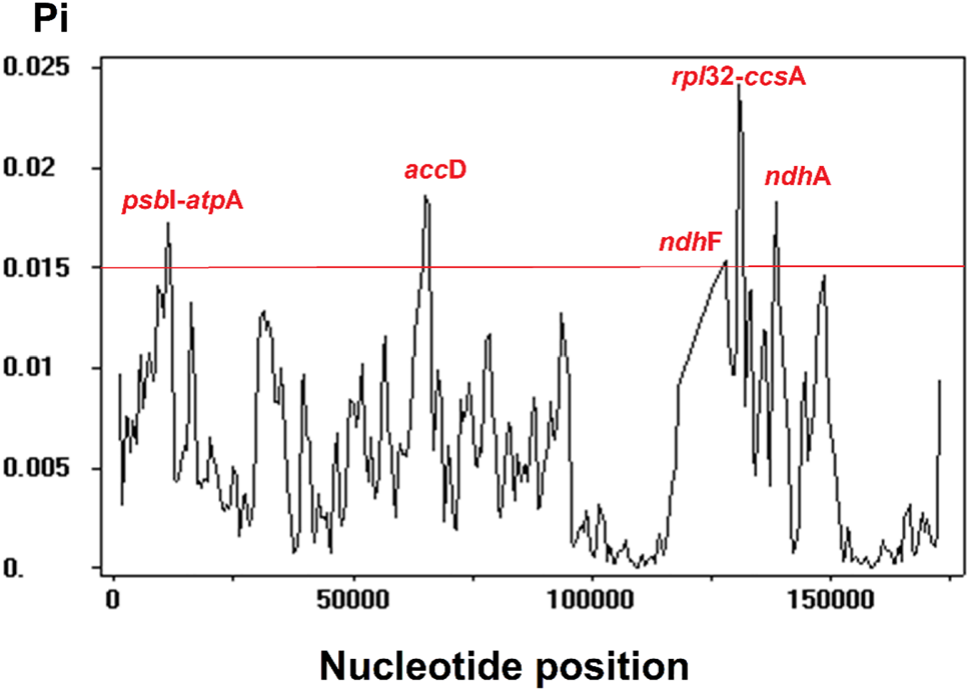
Highly variable regions in the complete chloroplast genome sequence of 31 selected *Ipomoea* taxa using a sliding window analysis (window length: 1000 bp; step size: 500 bp), with the cut-off point for the nucleotide diversity, Pi = 0.015. The x-axis and y-axis indicate the position of the midpoint of the nucleotide diversity of each window, respectively.

The sequence alignment of the 31 selected complete genome sequences of *Ipomoea* resulted in a total of 174,106 nucleotide sites, which expressed 139,750 and 5565 for invariable (monomorphic) and variable (polymorphic) sites, respectively. Among the variable sites, 2988 were singleton variable sites, and 2577 were parsimony informative sites. Within the singleton variable sites, 2930 contained two variants, while 58 contained three variants. For the parsimony informative sites, 2382 contained two variants; 192 contained three variants; and three contained four variants. By selecting Pi ≥ 0.015 as the cut-off point, five distinct highly variable regions, also known as nucleotide hotspots, were identified in the sequence alignment (Fig. 5). The first two regions were in the LSC region. The first region was located at sites 10,407–12,022, which is the intergenic spacer region *psb*I-*atp*A. The second region was placed between sites 62,705 and 66,381, which contained the *acc*D gene. The last three regions were located in the SSC region, at sites 125,097–128,643, 130,164–132,192, and 137,869–138,949, which contained the gene *ndh*F, the intergenic spacer region *rpl*32-*ccs*A, and the gene *ndh*A, respectively.

### Intraspecific variation between two *I. obscura* complete chloroplast genome sequences

The complete chloroplast genome of *I. obscura* from Taiwan contained 84 CDS, 37 tRNA, and eight rRNA genes. The pairwise distance between the two selected *I. obscura* complete chloroplast genome sequences was 0.000421. A total of 67 polymorphic sites were detected along the genome sequence, of which 43 were found in the LSC region, 16 were located in the SSC region, and eight were placed in the IR region.

### Phylogenetic relationship in *Ipomoea*

**Figure 6:**
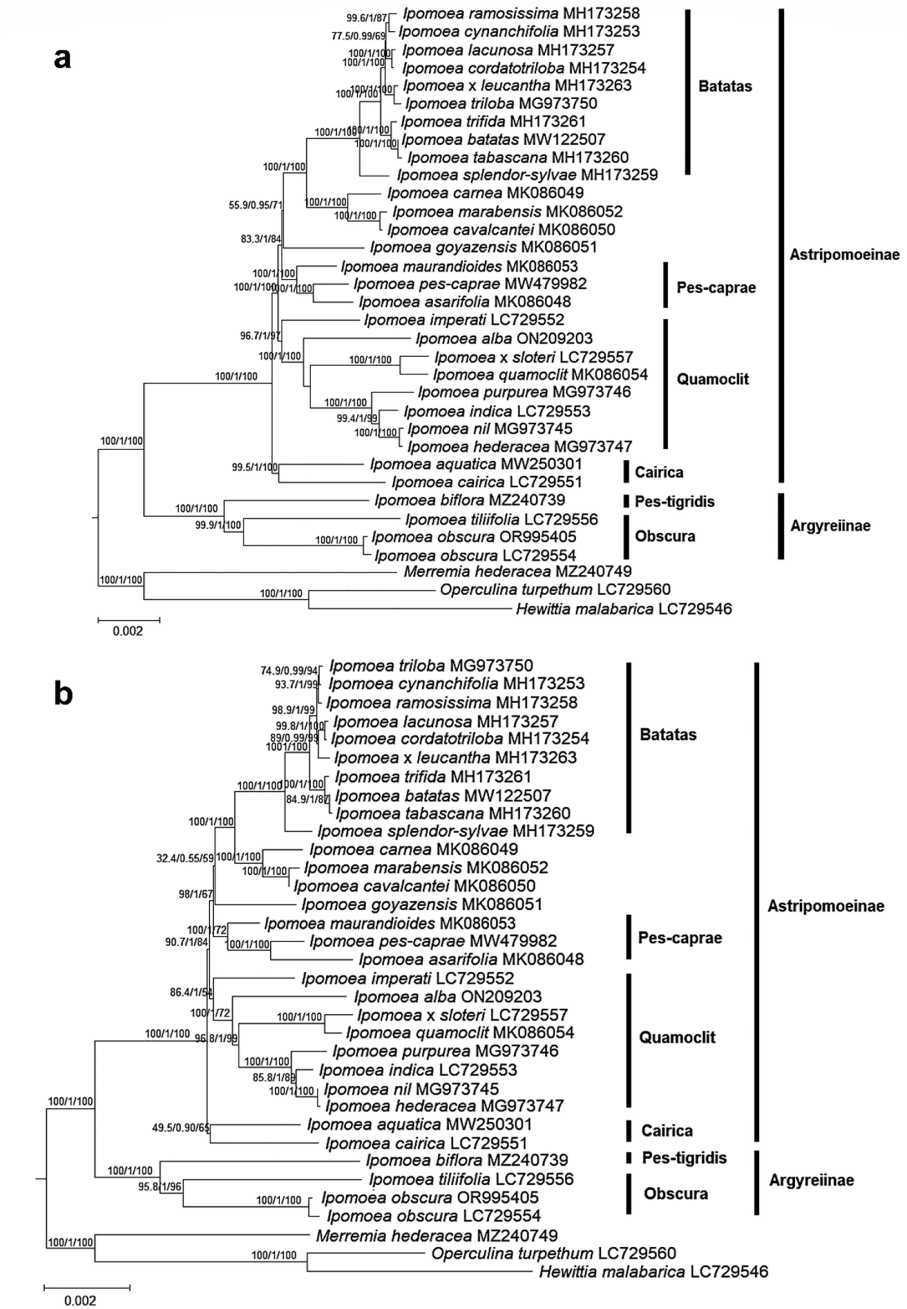
Phylogenetic analysis based on the 31 selected *Ipomoea* taxa in Thailand using the (**a**) complete chloroplast genome sequence, with the IRa sequence excluded, and (**b**) concatenated dataset of 76 shared unique CDS. Three closely related species from Merremieae, including *Hewittia malabarica* (GenBank accession no.: LC729546), *Merremia hederacea* (GenBank accession no.: MZ240749), and *Operculina turpethum* (GenBank accession no.: LC729560) were included as outgroup. A reliable branch support is indicated with an SH-aLRT support value (left) of ≥ 80%, a posterior probability (centre) of ≥ 0.95, and an ultrafast bootstrap support value (right) of ≥ 95%. The corresponding GenBank accession number is placed behind the name of each species. The classification for the clades and series level in Ipomoeeae that is indicated at the right side of the tree is as proposed by Eserman et al.^21^.

The phylogenetic tree based on the complete chloroplast genome sequence of 31 *Ipomoea* taxa and three other closely related outgroup taxa revealed a nearly resolved relationship within *Ipomoea*, in which a reliable branch support is indicated with an SH-aLRT support value (left) of ≥ 80%, a posterior probability (centre) of ≥ 0.95, and an ultrafast bootstrap support value (right) of ≥ 95% (Fig. 6a). Branch nodes that were not fully supported are the split between *I. cynanchifolia* + *I. ramosissima* and *I. lacunosa* + *I. cordatotriloba* (SH-aLRT = 77.5%, UFboot = 69%), the divergence of *I. goyazensis* (SH-aLRT = 55.9%, UFboot = 71%), and the divergence of the ser. Pes-caprae group (SH-aLRT = 83.3%, UFboot = 84%). In *Ipomoea*, *I. alba* is clustered with the other seven taxa, including *I. hederacea*, *I. imperati*, *I. indica*, *I. nil*, *I. purpurea*, *I. quamoclit*, and *I.* × *sloteri*; *I. imperati* was the first to diverge in this group, which was then followed by *I. alba*. For *I. obscura*, the two accessions were grouped together and are closely related to *I. tiliifolia*.

By using the 76 shared unique CDS dataset to reconstruct the phylogenetic tree of *Ipomoea*, the overall arrangement of the groups is congruent to those reconstructed using the chloroplast genome dataset, but with minor variation in molecular placement for some species (Fig. 6b). This includes the molecular placement of *I. triloba* in ser. Batatas. The weak branch supports identified in the chloroplast genome dataset remained unsupported at the CDS level, while some branch nodes in the chloroplast genome-based tree that are considered reliable, happened to be unsupported when using the CDS dataset, i.e. the divergence of *I. imperati* (SH-aLRT = 86.4%, UFboot = 54%).

### Molecular dating of *Ipomoe*a

**Figure 7:**
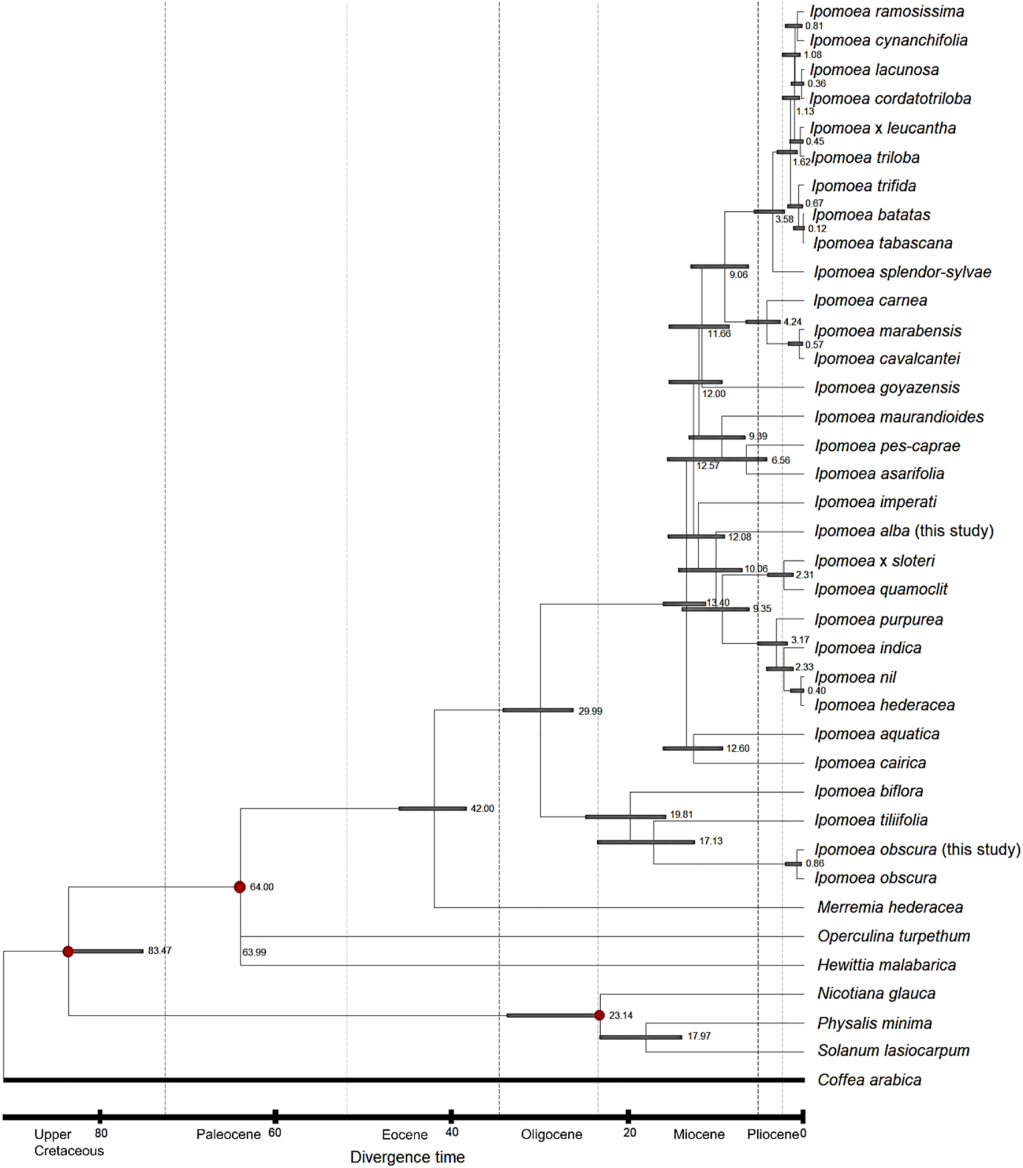
Time tree showing RelTime timescales of Ipomoeeae and Convolvulaceae inferred from the complete chloroplast genome sequence of 31 selected *Ipomoea* taxa in Thailand, three species of Merremieae, i.e. *Hewittia malabarica*, *Merremia hederacea*, and *Operculina turpethum*, and three species of Solanaceae, i.e. *Nicotiana glauca* (GenBank accession no.: MT985321) of Nicotianoideae as well as *Physalis minima* (GenBank accession no.: PP471919) and *Solanum lasiocarpum* (GenBank accession no.: PP234975) of Solanoideae. Red dots indicate the calibrated nodes. The corresponding 95% confidence of each estimated age is shown by a horizontal grey bar and the estimated age is indicated in millions of years. *Coffea arabica* (GenBank accession no.: EF044213) of Rubiaceae, Gentiales was selected as the outgroup.

Based on the three-time point calibrated time tree, the divergence between Convolvulaceae and Solanaceae was estimated to be around 83.47 Mya, and the split between Ipomoeeae and Merremieae was thought to happen around 42 Mya (Fig. 7). The mean age of the common ancestor of Ipomoeeae was calculated to be approximately 29.99 Mya, while the divergence of the two major clades, Astripomoeinae and Argyreiinae, was speculated to happen around 13.40 Mya and 19.81 Mya, respectively. For the two *Ipomoea* species used in this study, *I. alba* was calculated to diverge from the others of ser. Quamoclit around 10.06 Mya, while the split between *I. obscura* and *I. tiliifolia* was speculated to happen around 17.13 Mya. The divergence between the two *I. obscura* accessions was estimated to be around 0.86 Mya.

## Discussion and conclusion

The complete chloroplast genomes of *I. alba* and *I. obscura* displayed a conserved genome structure and size when compared to other published records of *Ipomoea* taxa. However, there was a difference in the number of genes between the two chloroplast genomes reported in this study when compared to the chloroplast genomes of selected *Ipomoea* taxa reported in literatures. The difference in the number of genes annotated might be due to the application of different annotation tools^40^. To identify the presence of such an error, we compared the gene content between the original annotation file that was annotated using Geneious Prime^48^ and the GeSeq-reannotated file of *I. obscura* (GenBank accession no.: LC729554). The original annotated file that was deposited in the GenBank database contains only 82 CDSs, which is two genes less than those annotated using GeSeq (Supplementary Table S4). On the other hand, most of the published chloroplast genome sequences of *Ipomoea* had a similar GC content, which is 37.5%. The GC content is believed to be a significant marker to identify the genetic relationship of species; closely related species should often contain highly similar GC content across them^49^. GC content in the IR region is usually higher when compared to the single-copy regions, which is contributed by the presence of tRNA and rRNA genes in that region^50^.

Both *I. alba* and *I. obscura* revealed different amounts of mono- and dinucleotide sequence repeats in their chloroplast genomes. Eventually, SSRs containing A or T units occurred more often when compared to those with C or G units, due to the overall AT richness in most chloroplast genomes^51^. Also, mononucleotide motifs are usually the most abundant repeat type, followed by dinucleotide motifs^52^. In some cases, SSRs from chloroplast genomes could serve as useful markers in the analysis of phylogenetic relationships or population genetics owing to their high polymorphism rates and consistency in reproducibility^53^. On the other hand, large repeats may play an important role in the events of gene arrangements and sequence divergence in the chloroplast genome^54^. Both the chloroplast genomes of *I. alba* and *I. obscura* shared the same number of large repeats detected. Although there is no evidence provided on the conserved variation between the two *Ipomoea* species in terms of repeat sequences, other species of *Ipomoea* were also reported to have a similar number of large repeats^24^. This suggests that the evolution rate of sequence repeats in *Ipomoea* could be consistent across all reported species thus far. For tandem repeat sequences, it is known to affect the genome structure in terms of genome size, arrangement, and gene duplication^55^. While small-sized tandem repeats (i.e. 10–39 bp) were detected in the two *Ipomoea* species, the two specific larger-sized tandem repeats that were detected in *I. alba* suggested that the chloroplast genome of *I. alba* is experiencing a more intense evolution rate via such an approach when compared to that of *I. obscura*.

Codon usage bias is a crucial element in the evolution of various genomes, principally influenced by the interaction between directional mutation pressure and natural selection^56^. Analysis through PR2 and neutrality plots revealed that codon preferences in *I. alba* and *I. obscura* are affected not only by mutations but also significantly by natural selection. This finding not only emphasizes the significance of natural selection in establishing codon usage bias but also correlates with studies in other species of *Ipomoea*, including *I. aquatica*, *I. batatas*, *I. nil*, *I. trifida*, and *I. triloba*^57^. The high similarity in the codon usage bias pattern in *Ipomoea* implies that these species would have encountered comparable environmental circumstances and selection pressures throughout their evolutionary trajectory.

The difference in the placement of the IR borders in the chloroplast genomes of the selected *Ipomoea* taxa could be a result of the expansion or contraction of the IR regions in the chloroplast genome. This was supported by the evidence where genes are detected crossing over the IR border, into or out of the IR region, showing events of gene flow and single-double gene transfer occurring at the four IR borders in the chloroplast genomes of angiosperms^49^. It was evident that events of expansion or contraction happen in the IR regions among members of Convolvulaceae, in which some CDSs (i.e. *rpl*2 and *rpl*23) that are usually located in the IR region were found in the LSC region^48^, while CDSs (i.e. *rps*15 and *ndh*F) were removed from the SSC region but occur in the IR region in duplicates^25^. The parallel expansion and contraction experienced by different species in Convolvulaceae, including *I. alba* and *I. obscura*, suggests that such evolution could be synapormorphic^25^.

When compared to the chloroplast genome sequence of *I. batatas*, the chloroplast genome sequence of *I. obscura* is more divergent than that of *I. alba*. The presence of indels is more abundant in the intergenic spacer regions, as the sequences in these regions are not encoded with protein sequences, making them easier to alter during the DNA repair process^58^. On the other hand, it is thought that intergenic spacer regions contain a high number of repeat sequences; while the repeat sequences could influence the genome size, the difference in the nucleotide substitution rate in these sequence repeats across these closely related species could have resulted in distinct areas of indels in *I. obscura* when compared to *I. alba* and *I. batatas*. Eventually, members of *Ipomoea* exhibited distinct indel variations in their chloroplast genome, which enabled them to be constructed into specific markers for species characterization. It was demonstrated that two indel markers were developed to authenticate *I. nil* and *I. purpurea* from other closely related species, as they hold significant medicinal value in traditional medicine production^23^.

Based on the genome sequence alignment of 31 *Ipomoea* taxa, the highly variable regions in the chloroplast genome sequence of *Ipomoea* are concentrated in the single-copy regions. The five hotspot regions identified in this study could be developed into gene markers that could properly delimit *Ipomoea* species. So far, phylogenetic analysis of short DNA sequences is only confined to the use of nuclear-based gene regions such as the nuclear ribosomal internal transcribed spacer region and the nuclear gene *waxy*^14^; plastid-based phylogenetic analysis of *Ipomoea* is generally lacking. Although the cost of next-generation sequencing has become affordable in many countries, making the effort to assemble the chloroplast genome sequence more convenient, as one of the diverse angiosperm genera, this effort could be cost-prohibitive. Thus, it is wise to identify highly polymorphic and relatively shorter chloroplast-based barcodes for *Ipomoea*. This is because the gene markers derived from the highly variable regions identified from the sliding window analysis could provide a better resolution in species delimitation for closely related species when compared to the universal DNA barcoding loci^59^. While it is recommended to have at least five to seven closely related chloroplast genome sequences from different species to identify such barcodes^60^, our study has utilized 31 chloroplast genomes derived from 30 different *Ipomoea* taxa, making the finding highly reliable when compared to other published works (i.e. Park et al.^23^, Wang et al.^61^). However, we found that, despite the smaller sampling size used, the findings for Refs.^23^ and^61^ are somewhat congruent with our finding, as well as those of Sun et al.^24^, who included complete chloroplast genome sequences of 40 *Ipomoea* taxa in the sliding window analysis. It is clear that the SSC region contains a significant amount of these highly variable regions in all studies. However, these hotspot regions need to be tested to confirm their efficacy to delimit *Ipomoea* species by reconstructing the neighbour-joining tree using either the three hotspot regions separately or in a combined dataset^62^.

When reconstructing the phylogenetic tree of closely related species, the complete chloroplast genome sequence data could be superior to the chloroplast CDS data because the former contains a significant amount of genetic information, as demonstrated in Ref.^60^. These informative sites will contribute to a better resolution in delimiting closely related species, revealing well-resolved phylogenetic relationships in complicated genera or families^63^. When compared to the nuclear-based phylogenetic tree, the molecular placement of *I. alba* is congruent to that using the complete chloroplast genome sequence; *I. alba* is also strongly resolved as the species to diverge before *I. hederacea*, *I. purpurea*, and *I. quamoclit* when using the combined dataset of ITS and *waxy* sequences^14^, and is placed in the same group as *I. hederacea*, *I. indica*, *I. nil*, and *I. purpurea*, being the first to diverge among the five species under ser. Quamoclit, based on the phylogenetic tree reconstructed using 605 putative single-copy nuclear regions^64^. However, as the clade represented by *I. biflora* , *I. tiliifolia*, and *I. obscura* should contain some other species derived from Argyreiinae^21^, based on the limited sampling size used in this study, we can only verify that *I. obscura* is grouped with *I. tiliifolia* when analysed using the ITS sequences based on the finding by Ref.^15^. To have a clearer view on the molecular placements of these species at the nuclear level, an attempt to reconstruct the ML tree using the ITS sequence was carried out using IQ-tree based on the published sequence data available in the GenBank database. The best-fit model according to BIC for the 34-taxa sequence dataset was the TIM2 model with equal base frequencies (TIM2e) + G4 (= TIM2e + G4). Generally, the findings from the complete chloroplast genome sequences still complement those of the short nuclear gene sequence datasets (i.e., ITS), despite minor conflicts in species placements that are noticeable at the series level (see Supplementary Fig. S3). For example, *I. aquatica* and *I. cairica* are not in the same clade, despite both belonging to ser. Cairica; *I. biflora* is clustered with I. *obscura* and *I. tiliifolia* of ser. Obscura, despite belonging to ser. Pes-tigridis. The difference in topology can be explained by the presence of poorly supported branch nodes due to the limited informative sites in these selected gene sequences. Cyto-nuclear discordance is a common issue in some angiosperms^65,66^. There are many reasons to explain the presence of conflict in lineage sorting, for example, hybridization and cytoplasmic introgression^67^. However, to better explain the phylogenetic relationship among species of *Ipomoea*, a larger sampling size should be considered. Nevertheless, the genomic data generated in this study could serve as useful information for future studies on the species identification, classification, and evolutionary history of *Ipomoea*.

To date, there are at least two published works that attempt to put the diversification of *Ipomoea* within a temporal context with a divergence time analysis of Convolvulaceae using chloroplast data. The first study utilised the complete chloroplast genome sequences derived from 31 taxa of Ipomoeeae to reconstruct the time-calibrated phylogenetic tree. Similar to our work, three nodes were calibrated, in which the first time point was pointed to the crown group of Solanaceae, the second time point was applied to the stem group of Convolvulaceae, and the last time point was placed at the root of the most recent common ancestor of Merremieae; however, using recognized fossil pollen^21^. When compared to our findings, the mean ages of Ipomoeeae, Argyreiinae, and Astripomoeinae obtained from that study were somewhat older than those calculated in this study, which were around 35 Mya, 26 Mya, and 23 Mya, respectively. Eventually, this would raise the incongruity that the divergence of Ipomoeeae in Convolvulaceae that was previously thought to happen during the Eocene, could have happened later during the Oligocene. While there are no useful fossils available at present to properly calibrate the internal nodes within Ipomoeeae, we believe that our estimations would be more robust by having the same calibration time points obtained from TimeTree, of which the time estimates present in the database consist of the compilation of published molecular dating from a diverse species collection. It is also known that the TimeTree database is especially useful for sample groups that have little or no fossil record^68^. The divergence time between Argyreiinae and Astripomoeinae, when compared to the earlier study, also noted a change in epoch, during which the split between the two clades that was supposed to happen in the Oligocene, was speculated to occur during the Miocene in our study. Despite the long divergence time between the two clades, no distinct morphological characteristics could distinguish them accurately, suggesting that a process of parallel evolution could be happening in Ipomoeeae^21^.

On the other hand, the second study that performed molecular dating of *Ipomoea* utilized a five-gene chloroplast dataset, including the *atp*B, *mat*K, *ndh*F, *rbc*L, and the intergenic spacer *trn*L-*trn*F^69^. Instead of using fossil records to date their phylogenetic tree, the authors assumed the divergence time between Convolvulaceae and Solanaeceae by trying four different age estimates. As a result, the inferred crown ages for the two clades, Argyreiinae and Astripomoeinae, were speculated to be approximately 21 Mya and 25.6 Mya, respectively. Members of Aryreiinae are thought to be derived from the Old World, while Astripomoeinae are from the New World. Unlike the previous finding^21^ and our study, the divergence time based on the five-gene chloroplast dataset speculated that the Old-World clade was estimated to happen later than the New World clade. We supposed that the age of the divergence time estimates could be affected by the sampling size used in each study, since the age of the two clades is determined based on the split of the basal group of the mentioned clade. However, the divergence between *I. batatas* and *I. trifida* was inferred to have occurred at least 0.89 Mya in the chloroplast time-calibrated phylogeny, which is slightly older but still within the estimated range of our finding, i.e. 0.67 Mya, during the Pleistocene. It is anticipated that due to the differences in calibrated time points and sampling size, variation in age estimates will be present^45,70^. Yet, the inclusion of CI estimates from TimeTree could provide greater accuracy in divergence time estimation for big data when using the ML-based RelTime analysis, in which the estimates are usually closer to the true generating age of nodes through a fuzzy method^71^. Thus, the divergence time for *Ipomoea* estimated in this study is deemed to be fairly accurate, assuming that all branch supports are credible and the calibration is correct.

### Supplementary Information

Supplementary Figure S1.

Supplementary Figure S2.

Supplementary Figure S3.

Supplementary Table S1.

Supplementary Table S2.

Supplementary Table S3.

Supplementary Table S4.

Supplementary Legends.

## Data Availability

The data that support the findings of this study are openly available in GenBank of NCBI at https://www.ncbi.nlm.nih.gov, accession number (ON209203 and OR995405). The raw NGS data that support the findings of this study are available from the corresponding author, A.C., upon reasonable request.
