# Supplementary figures and images for: The chloroplast genome sequences of Ipomoea alba and I. obscura (Convolvulaceae): genome comparison and phylogenetic analysis
Source: Sci Rep. 2024 Jun 18;14:14078. doi: 10.1038/s41598-024-64879-8 (PMC11189557; doi:10.1038/s41598-024-64879-8)

**a**  
Depth

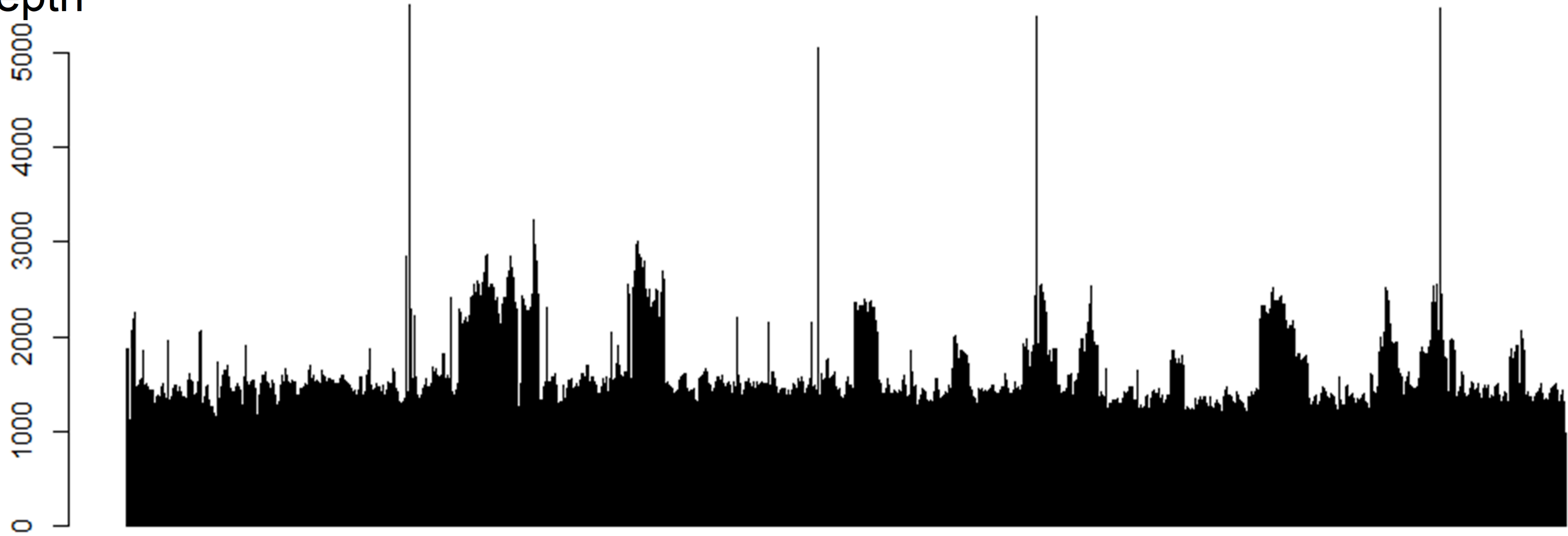

Position

**b**  
Depth

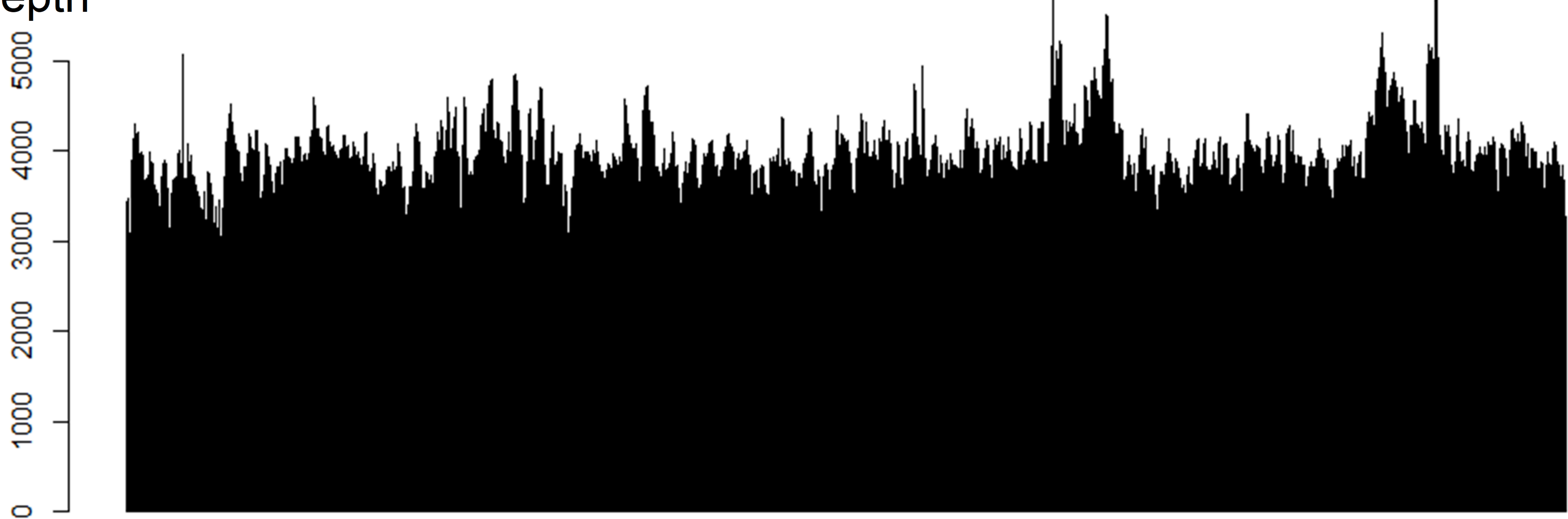

Position

Supplement: Supplementary file 1 — Supplementary Figure S1. [file 41598_2024_64879_MOESM1_ESM.pdf]

(a)

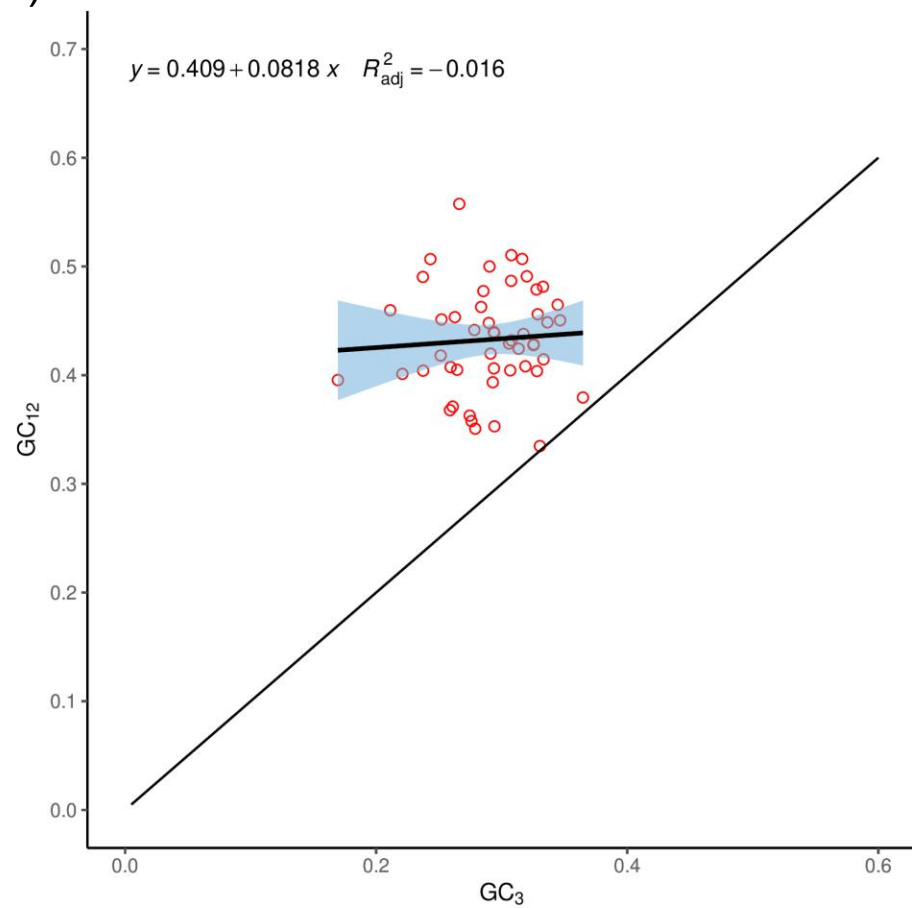

(b)

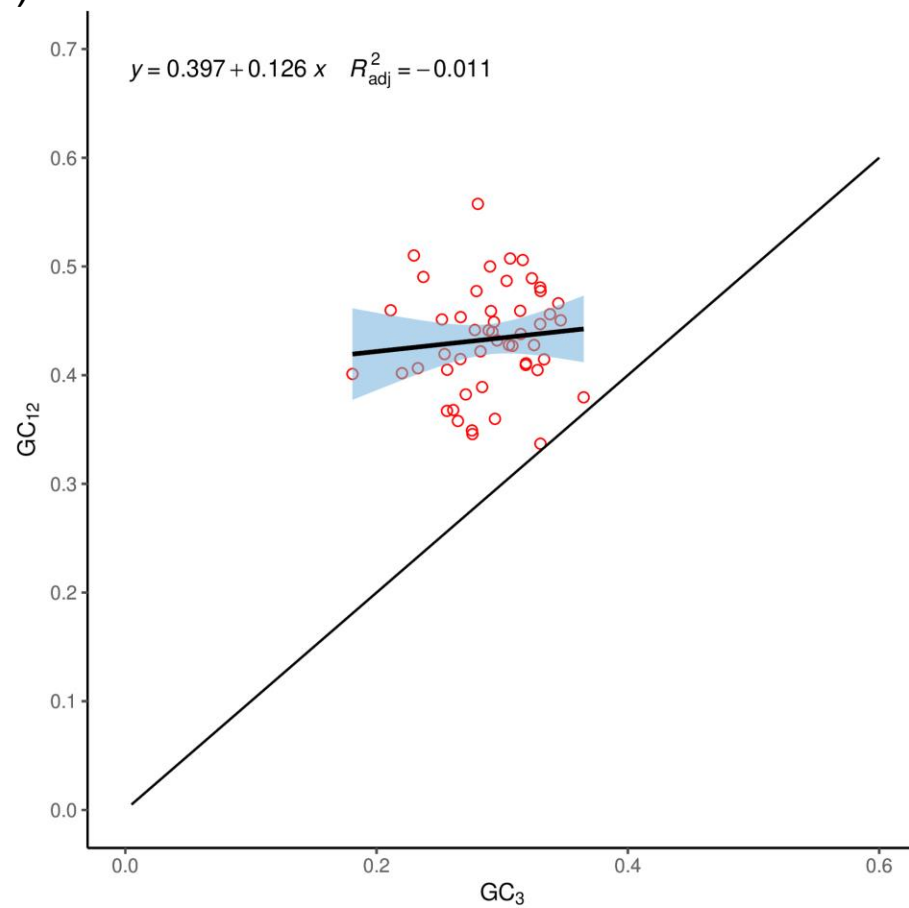

Supplement: Supplementary file 2 — Supplementary Figure S2. [file 41598_2024_64879_MOESM2_ESM.pdf]

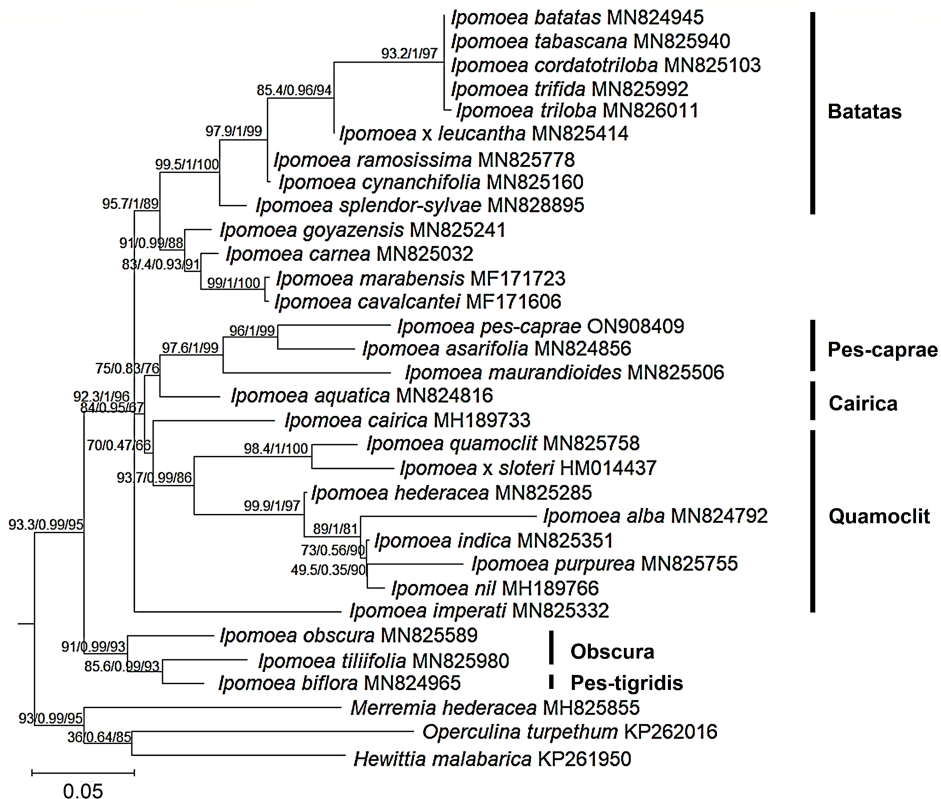

Supplement: Supplementary file 3 — Supplementary Figure S3. [file 41598_2024_64879_MOESM3_ESM.pdf]
