# Supplementary material for: The chloroplast genome sequences of Ipomoea alba and I. obscura (Convolvulaceae): genome comparison and phylogenetic analysis
Source: Sci Rep. 2024 Jun 18;14:14078. doi: 10.1038/s41598-024-64879-8 (PMC11189557; doi:10.1038/s41598-024-64879-8)
Supplement: Supplementary file 4 — Supplementary Table S1. [file 41598_2024_64879_MOESM4_ESM.pdf]

No.	Species	GenBank accession number
1	*Ipomoea alba*	ON209203 (this study)
2	*Ipomoea aquatica*	MW250301
3	*Ipomoea asarifolia*	MK086048
4	*Ipomoea batatas*	MW122507
5	*Ipomoea biflora*	MZ240739
6	*Ipomoea cairica*	LC729551
7	*Ipomoea carnea*	MK086049
8	*Ipomoea cavalcantei*	MK086050
9	*Ipomoea cordatotriloba*	MH173254
10	*Ipomoea cyanchifolia*	MH173253
11	*Ipomoea goyazensis*	MK086051
12	*Ipomoea hederacea*	MG973747
13	*Ipomoea imperati*	LC729552
14	*Ipomoea indica*	LC729553
15	*Ipomoea lacunosa*	MH173257
16	*Ipomoea marabensis*	MK086052
17	*Ipomoea maurandioides*	MK086053
18	*Ipomoea nil*	MG973745
19	*Ipomoea obscura*	LC729554
20	*Ipomoea obscura*	OR995405 (this study)
21	*Ipomoea pes-caprae*	MW479982
22	*Ipomoea purpurea*	MG973746
23	*Ipomoea quamoclit*	MK086054
24	*Ipomoea ramosissima*	MH173258
25	*Ipomoea splendor-sylvae*	MH173259
26	*Ipomoea tabascana*	MH173260
27	*Ipomoea tiliifolia*	LC729554
28	*Ipomoea trifida*	MH173261
29	*Ipomoea triloba*	MG973750
30	*Ipomoea* x *leucantha*	MH173263
31	*Ipomoea* x *sloteri*	LC729552
