# Supplementary material for: The chloroplast genome sequences of Ipomoea alba and I. obscura (Convolvulaceae): genome comparison and phylogenetic analysis
Source: Sci Rep. 2024 Jun 18;14:14078. doi: 10.1038/s41598-024-64879-8 (PMC11189557; doi:10.1038/s41598-024-64879-8)
Supplement: Supplementary file 7 — Supplementary Table S4. [file 41598_2024_64879_MOESM7_ESM.pdf]

**Group of function**	**List of protein-coding genes**
Large subunit of ribosome	*rpl*2, *rpl*14, *rpl*16, *rpl*20, *rpl*22, *rpl*23, *rpl*32, *rpl*33, *rpl*36
Small subunit of ribosome	*rps*2, *rps*3, *rps*4, *rps*7(×2), *rps*8, *rps*11, *rps*12, *rps*14, *rps*15(×2), *rps*16, *rps*18, *rps*19
DNA-dependent RNA polymerase	*rpo*A, *rpo*B, *rpo*C1, *rpo*C2
Photosystem I	*psa*A, *psa*B, *psa*C, *psa*I, *psa*J
Photosystem II	*psb*A, *psb*B, *psb*C, *psb*D, *psb*E, *psb*F, *psb*H, *psb*I, *psbJ*, *psb*K, *psb*L, *psb*M, *psb*T, *psb*Z
NADH dehydrogenase	*ndh*A, *ndh*B(×2), *ndh*C, *ndh*D, *ndh*E, *ndh*F, *ndh*G, *ndh*H, *ndh*I, *ndh*J, *ndh*K
Cytochrome b6/f complex	*pet*A, *pet*B, *pet*D, *pet*G, *pet*L, *pet*N
ATP synthase	*atp*A, *atp*B, *atp*E, a*tp*F, *atp*H, *atp*I
RubisCO	*rbc*L
Photosystem assembly factors	*paf*I, *paf*II
Photosystem biogenesis factor	*pbf1*
Maturase	*mat*K
ATP-dependent protease	*clp*P1
Envelope membrane protein	*cem*A

Acetyl-CoA-carboxylase	*acc*D
Translational initiation factor 1	*inf*A
C-type cytochrome synthesis	*ccs*A
Hypothetical chloroplast reading frames	*ycf*1(×2), *ycf*2(×2)

Note: (×2) = comes with duplicates.
