# Supplementary material for: The chloroplast genome sequences of Ipomoea alba and I. obscura (Convolvulaceae): genome comparison and phylogenetic analysis
Source: Sci Rep. 2024 Jun 18;14:14078. doi: 10.1038/s41598-024-64879-8 (PMC11189557; doi:10.1038/s41598-024-64879-8)
Supplement: Supplementary file 8 — Supplementary Legends. [file 41598_2024_64879_MOESM8_ESM.docx]

**Supplementary material**

**Supplementary Table S1** GenBank accession numbers of the 31 selected *Ipomoea* taxa used in this study.

**Supplementary Table S2** Relative synonymous codon usage of protein-coding genes in *I.* *alba* and *I.* *obscura*.

**Supplementary Table S3** Parity Rule 2 plot analysis of protein-coding genes in *I.* *alba* and *I.* *obscura*.

**Supplementary Table S4** List of annotated protein-coding genes in *Ipomoea* *obscura* chloroplast genome (GenBank accession no. LC729554) using GeSeq v2.03.

**Supplementary Figure S1** Genome depth coverage analysis of the assembly result for the complete chloroplast genome sequence of (a) *I. alba* and (b) *I. obscura*.

**Supplementary Figure S2** Neutrality plot for genes of the chloroplast genome of (a) I. alba and (b) I. obscura.

**Supplementary Figure S3** Phylogenetic tree reconstructed using the nuclear ribosomal internal transcribed spacer (ITS) sequence of 30 selected *Ipomoea* species in Thailand, downloaded from the GenBank database.
